# Among- and within-population variation in germination response shapes ecological resilience in the Mediterranean cliff species *Brassica incana*

**DOI:** 10.1093/aob/mcae172

**Published:** 2024-10-14

**Authors:** Lucrezia Laccetti, Diana María Cruz Tejada, Alessio Mo, Angelino Carta, Giovanni Scopece

**Affiliations:** Department of Biology, University of Naples Federico II, Naples, Italy; Department of Biology, University of Pisa, Pisa, Italy; Department of Biology, University of Pisa, Pisa, Italy; Department of Biology, University of Pisa, Pisa, Italy; Department of Biology, University of Naples Federico II, Naples, Italy

**Keywords:** *Brassica incana*, climate change, genotype-by-environment interaction, local adaptation, seed germination

## Abstract

**Background and Aims:**

Understanding how plant species respond to extreme conditions is crucial for predicting their ecological resilience under climate change. Here, we aimed to forecast the ecological resilience of the Mediterranean cliff species *Brassica incana* (Brassicaceae) by estimating population variation in germination response under novel extreme environmental conditions.

**Methods:**

We investigated the thermal germination responses in 14 populations of *B. incana* by exposing seeds to temperatures within and outside conditions experienced in their local environment. Then, we quantified among- and within-population variation in germination response to extreme temperatures, estimated genotype-by-environment interactions (G × E) and tested if population performance at extreme temperatures is explained by local climate.

**Key Results:**

We found significant among-population differences in germination response, a different level of within-population variability and different mechanisms underlying G × E patterns. Also, populations experiencing higher temperatures in their local environment showed a better performance at both cold and hot extremes while populations experiencing lower temperatures showed a limited ability to germinate under extreme conditions.

**Conclusions:**

Our results suggest that populations experiencing higher temperatures in their local environment have a greater potential to face future thermal extreme conditions and their role is thus crucial to promote species ecological resilience.

## INTRODUCTION

Ongoing climate change is a major threat to the future persistence of plant species since it will alter environmental conditions and thus expose plants to novel conditions, outside their current niche (hereafter extreme conditions). Hence, understanding how plant species can respond to such conditions is crucial for predicting their ecological resilience under a climate change scenario (Edwards and Richardson, 2004; Thuiller, 2004).

Species ecological resilience is expected to increase if there is intra-specific variation in population responses to environmental changes with some populations being more prone to persist than others (Barga *et al.*, 2017; Walter *et al.*, 2020). Population persistence under a climate change scenario can be achieved through migration towards more suitable environments (Pearson and Dawson, 2003) or through local adaptation, with both scenarios being highly dependent upon the species distribution (Chmielewski and Rötzer, 2001, Angert *et al.*, 2011). Indeed, species characterized by a highly fragmented distribution may have limited chance to shift or expand their geographical range in response to climate change (Opdam and Wascher, 2004) and thus *in situ* responses play a crucial role in determining their future persistence. Also, reduced gene flow among populations can increase the chances for local adaptation which can be a driver of among-population variation in their environmental sensitivity (*sensu*Walter *et al.*, 2020). For instance, populations experiencing high local temperatures might be pre-adapted to successfully face higher temperatures predicted under a climate change scenario (Walter *et al.*, 2020). Alternatively, differences among populations can arise as a consequence of random genetic changes (e.g. drift) and populations can exhibit different abilities to face novel conditions, regardless of the environment in which they are tested (Kawecki and Ebert, 2004). Under the first scenario, an interaction between population and environment, known as genotype-by-environment interaction (G × E; Kawecki and Ebert, 2004; Anderson *et al.*, 2011; Josephs, 2018), can be hypothesized suggesting that population response is more predictable under specific environmental conditions. In this context, experiments in which plant performance is assessed under various environmental conditions, within and outside their current conditions, represent a particularly powerful tool to assess whether population performance is affected by specific environmental conditions or if different populations show equivalent performances under multiple environments (Hargreaves *et al.*, 2014).

Mean population response can provide important information; however, it is crucial to take into account potential within-population variations by assessing the performance of different genotypes or related genotypes (e.g. half-sib or full-sib progenies) under a wide range of environmental conditions. Such variations can be explained by several genetic and environmental factors (e.g. maternal effects, additive genetic variance or environmental heterogeneity) and can thus underpin different levels of ecological resilience. For instance, maternal effects can contribute to within-population variation since maternal provisioning can help offspring cope with environmental changes (Donohue and Schmitt, 1998; Galloway, 2005), while fluctuating environmental factors at small temporal or spatial scales can impose variable selection hence maintaining a high level of variance within populations (Cohen, 1966; Christiansen, 1974; Venable, 2007; Delph and Kelly, 2014; Friedman *et al.*, 2019).

Seed germination is an early life-stage transition of plants, setting the context for subsequent development and natural selection (Donohue *et al.*, 2010). Therefore, germination modelling is crucial to examine the joint effect of environmental and genetic factors in shaping population variance under extreme conditions (Donohue *et al*., 2010; Walter *et al.*, 2020; Carta *et al.*, 2022) and thus to predict population persistence and resilience in response to climate change (Walck *et al.*, 2011; Cochrane, 2016; Baskin and Baskin, 2022; Mattana *et al.*, 2023). Indeed, early development phases of plant life cycles such as germination are probably the most vulnerable under a climate change scenario, often suffering the highest mortality and thus representing a critical event for plants. For this reason, germination modelling of plant responses to climate changes has rapidly increased in the last decade (Picciau *et al.*, 2019; Sentinella *et al.*, 2020; Mattana *et al.*, 2023; Price *et al.*, 2024), especially in regions predicted to be more sensitive to climate modifications, such as the Mediterranean (Giorgi and Lionello, 2008; Lionello and Scarascia, 2018; Mattana *et al.*, 2022).

The ongoing climate change in the Mediterranean is likely to generate warmer climates and hence Mediterranean plants, in order to track rain availability, may expose seeds to temperatures outside their current local germination conditions (Giorgi and Lionello, 2008; Lionello and Scarascia, 2018). These climate variations are expected to greatly affect the timing and success of germination since many species rely on specific climatic patterns to break seed dormancy and trigger germination (Donohue *et al.*, 2010 and references therein; Walck *et al.*, 2011). Under this scenario, populations that respond positively both to cold and hot extremes have a higher potential to persist under novel changing environmental conditions. Understanding *in situ* responses is particularly important in the Mediterranean since its landscape fragmentation can severely limit the chance of plant populations to migrate in response to climate change (Benito Garzón *et al.*, 2019; Muffler *et al.*, 2021; Mattana *et al.*, 2022). Therefore, the Mediterranean represents a useful study system to test plants’ ability to persist in their local environment and estimate their ecological resilience.

Here, we used as a model system *Brassica incana* (Brassicaceae), a Mediterranean cliff species characterized by a highly fragmented distribution, with limited chance to expand or shift its distribution in response to climate change and with a strong genetic population structuring (Ciancaleoni *et al.*, 2018). We investigated the variation in its ability to face extreme temperatures across 14 populations located in Southern Italy and Sicily and across different maternal families within populations. To do this, we performed a laboratory germination experiment where seeds from each maternal family were exposed to different germination temperatures representing conditions within and outside the range of conditions experienced by populations in their local environment. Population germination responses were then correlated to local temperature conditions to test if local climate is a driver of among-population variation in germination response to extreme temperatures.

Specifically, we addressed the following questions:

(1) Does germination response to extreme temperatures vary among *B. incana* populations?
(2) Does germination response to extreme temperatures vary within *B. incana* populations?
(3) Is local climate associated with population germination response to extreme temperatures?

## MATERIALS AND METHODS

### Study species and seed collection

*Brassica incana* Ten. is a wild relative of the *Brassica oleracea* L. crop native to Eastern and Southeastern Europe, mainly distributed along coastal cliffs of Southern Italy and Sicily and with a highly fragmented distribution (Snogerup *et al.*, 1990). It is a perennial species with a generalist pollination system (Frachon *et al.*, 2023) which produces cylindrical, bent siliques with rounded seeds inside (Snogerup *et al.*, 1990).

**Fig. 1.**
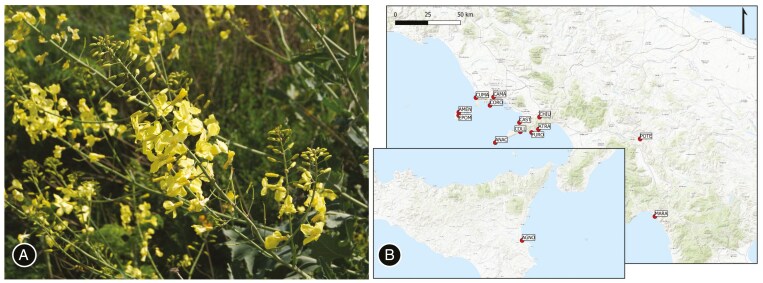
*Brassica incana* (A) and sampling locations of the 14 investigated populations (B).

To investigate how different populations diverge in their germination response to extreme temperature treatments, in early summer 2023, we collected 5908 seeds from 14 populations located in Southern Italy and Sicily experiencing different environmental conditions in their natural sites (Fig. 1; Supplementary Data Table S1). To take into account variability within populations, we collected seeds from fully ripened fruits from two to eight maternal families per population (inter-family distance ≥2 m) within an area of ~250 m^2^. This experimental design allows minimizing the amount of genetic variance compared to a design where seeds with unknown parental origin are tested and is widely used to characterize the adaptive potential of natural populations for which controlled crosses are difficult to manage *in situ* (Falconer and Mackay, 1996; Gauzere *et al.*, 2013 and references therein). Moreover, due to the distribution of *B. incana* and the pollinator behaviour, it is likely that most pollination occurs within a locality and thus that maternal families represent local genotypes (Frachon *et al.*, 2023).

Fruits collected from each maternal family were manually processed in the laboratory to extract seeds that were either used within 3 weeks after collection to examine the degree of seed dormancy at dispersal or after 6 months of dry after-ripening (at ~20 °C, 40% relative humidity). This timespan between the two experiments mimics the natural conditions experienced by seeds in the summer season, necessary to overcome seed dormancy in most Mediterranean species (Baskin and Baskin, 2014; Picciau *et al.*, 2019; Carta *et al.*, 2022) and thus ensuring the expression of the maximum germination potential of the species. This allows for properly modelling seed thermal responses within and outside the range of conditions experienced by populations in their local environment (i.e. extreme conditions; see below for further details in the experimental design).

### Experimental design

We set up a first germination experiment to investigate the overall seed germination responses to average temperature, temperature regimes and dry after-ripening (i.e. the extent of primary seed dormancy) in *B. incana* populations. For this reason, freshly collected and after-ripened seeds were exposed to optimal germination temperatures for most Mediterranean plant species (i.e. ~15 °C, Thanos *et al.*, 1995; Carta *et al.*, 2022) under two different temperature regimes, i.e. constant and alternating. Specifically, we used three constant temperatures (15, 20, 25 °C) and three alternating temperatures (20/10, 25/15, 30/20 °C). The overall effects of temperature, temperature regime and after-ripening on germination response were modelled using binomial generalized mixed models with Bayesian estimation as described in Supplementary Data Methods S1.

To investigate the germination response under extreme temperatures, we set up a second germination experiment using after-ripened seeds exposed to the same above-mentioned average temperatures as well as extreme temperatures (i.e. temperatures outside the range of conditions experienced by populations in their local environment and outside most optimal germination conditions; Dürr *et al.*, 2015; Mattana *et al.*, 2023). This experiment was conducted using after-ripened seeds under alternating extreme temperatures since our germination modelling (Supplementary Data Methods S1) revealed a significant positive overall effect of after-ripening (indicating the presence of seed dormancy in fresh seeds) and of the alternating temperature regime on seed germination (Table S2; Fig. S1). We used this approach since our main goal was to quantify germination response under extreme conditions and because an overall decrease in germination due to a lower sensitivity to constant temperatures could have biased our interpretation of population performance under extreme conditions.

To identify extreme temperatures for *B. incana* populations, we first retrieved autumn and summer mean temperatures (i.e. climate conditions experienced by seeds between dispersal and germination) from the ClimateEU database v.4.63 at 1.25 arc-min resolution for all the investigated populations, following the methodology described in Hamann *et al.* (2013). According to these data, populations experience different temperatures in both seasons with mean summer temperatures ranging from 21.51 to 25.90 °C, and mean autumn temperatures ranging from 15.01 to 19.33 °C (Supplementary Data Fig. S2). Then, we selected temperatures outside the range of conditions experienced by populations in their local environment (i.e. 10/5 and 35/30 °C) to investigate germination response to extreme temperatures.

In the two experiments, seeds were kept separately for each maternal family and placed on 9-cm-diameter plastic 1 % agar Petri dishes. We used from four to ten seeds for each maternal family in each treatment with an average number of eight seeds per family per treatment thus leading to an average of 171 seeds per population in the first experiment and an average of 256 seeds per population in the second experiment (at least three Petri dishes were used to replicate each treatment and each population). Petri dishes were randomly allocated to different temperature- and light-controlled incubators. Germination, i.e. the emergence of a root >2 mm, was checked every week for the whole duration of each experiment (30 d). Germination success was recorded as a binary trait (0 = failed to germinate; 1 = seed germinated) and subsequently analysed following binomial statistics. Since among-population variation in seed viability might influence our analyses, at the end of the experiments, cut-tests were conducted to evaluate the viability of ungerminated seeds, and dead seeds, as well as maternal families unable to germinate under all the tested temperatures, were removed from the analyses.

### Statistical analyses

Since fresh seeds exhibited some degree of dormancy and alternating regime showed a significant positive effect on germination response (Supplementary Data Methods S1; Table S2; Fig. S1), all the subsequent statistical analyses aiming to quantify germination sensitivity under extreme temperatures were performed using after-ripened (non-dormant) seeds exposed to alternating temperatures. Germination success was analysed using binomial generalized mixed models with Bayesian estimation using the R package *MCMCglmm* (Hadfield, 2010). All analyses were implemented using R v.4.3.2 (R Core Team, 2019).

The extent of variance among different maternal families can be the result of four putative sources of variation: (1) genetic maternal effects, (2) maternal environmental effects, (3) additive genetic variance and (4) non-additive genetic variance (i.e. dominance and epistasis; Lynch and Walsh, 1998; Völler *et al.*, 2012; Sober and Ramula, 2013). Therefore, although our experimental design based on maternal families does not allow disentangling the role of each component, to exclude a role of maternal resource provisioning in shaping germination patterns, we tested for an association between seed mass and germination response by performing a regression analysis between the mean seed mass of each maternal family (three replicates of 20 seeds per family) and its germination response. This approach is commonly used to account for maternal resource provisioning effects as it allows associating maternal provisioning and traits expressed at early life stages (e.g. Friedman *et al.*, 2019).

### Among-population variation in germination sensitivity at extreme temperatures

To investigate the germination response of after-ripened seeds under the two extreme temperatures, we used a character state approach where temperature was used as a discrete variable (see Walter *et al.*, 2020). By using this approach, we could estimate variance at specific conditions, as well as changes in variance and correlations between high and low temperatures. This represents a particularly useful approach to test for a genotype-by-environment interaction (G × E) at extreme temperatures (Walter *et al.*, 2020 and references therein).

We thus tested for among-population variation in germination sensitivity by modelling the germination response of each population across the two extreme treatments in a Bayesian framework which allowed determining whether G × E was the result of changes in variance in germination response or was explained by correlations across the environments. To do this, we performed a generalized linear mixed model:


$$\begin{array}{l} y_{ijklm}=T_{i}+p_{j(i)}+m_{k(i)}+d_{l(i)}+e_{m(ijkl)} \end{array}$$ (1)

where *T*_*i*_ is the extreme temperature, included as a fixed effect, *p*_*j(i)*_, *m*_*k(i)*_ and *d*_*l(i)*_ are the population, the maternal family and the Petri dish, respectively, included as a random factor, and *e*_*m(ijkl)*_ is the model error. From the model described in eqn (1), we estimated the covariance matrix of the random factor population by specifying an unstructured covariance matrix to estimate population-specific random intercepts for each temperature. The *n* × *n* covariance matrix (where *n* is the number of treatments) represents the among-population variance within each temperature and the among-population covariance between each temperature pairwise comparison. G × E is verified when there is a negative correlation between different temperature treatments, which suggests a trade-off in population germination response, or when variance differs between the treatments. We implemented eqn (1) using the same methodology described in Supplementary Data Methods S1. Briefly, we used 3 000 000 Markov chain Monte Carlo (MCMC) sampling iterations, with a burn-in period of 300 000 iterations and a thinning interval of 1000 iterations. Then, we extracted 1000 MCMC iterations from the model output, which provided the posterior distribution for the subsequent analyses. We also extracted the Best Linear Unbiased Predictors (BLUPs) for the population response to each temperature which were then used to visualize G × E interactions. To strengthen this analysis and test for a significant interaction between population and temperature, we also analysed the data using the *glmmTMB* package (Brooks *et al.*, 2017) by including population and temperature as random slopes.

### Within-population variation in germination sensitivity at extreme temperatures

The same approach used to test for significant G × E among the populations was also used to test for G × E within populations with at least five maternal families (*n*_populations_ = 4). These models were built separately for each population and the maternal family was used as a random factor. Covariance matrices were built as previously described for the random factor population and BLUPs were used to visualize the results. As described for the among-population comparison, we also analysed the data with the *glmmTMB* package by including maternal family and temperature as random slopes.

This approach could not be used for populations with fewer than five maternal families since random effects can only be used with sample sizes of at least five (Bolker *et al.*, 2009). Therefore, for the remaining populations (*n* = 8), we used maternal family as a fixed effect in the generalized linear mixed effect model:


$$\begin{array}{l} y_{ijkl}=T_{i}+M_{j}+T_{i}\times \;M_{j}+d_{k}+e_{l(ijk)} \end{array}$$ (2)

where *T*_*i*_ and *M*_*j*_ are the temperature and the maternal family, respectively, included as fixed effects, *d*_*k*_ is the Petri dish which was used as a random factor and *e*_*l(ijk)*_ is the model error. Here, G × E significance was tested as the interaction between maternal family and temperature within each population. For two populations (EPOM and POTE) we could not perform this analysis due to the lack of family replicates.

### Association between local climate and germination response to extreme temperatures

Lastly, to verify if germination response to extreme temperatures was driven by local climate, we compared germination success with climate data gathered from the ClimateEU database. To do this, we assessed the association between mean summer and mean autumn temperatures (i.e. temperatures to which seeds are typically exposed) and the relative germination performance at high and low extreme temperatures for each population using linear regression in the *lme4* package (Bates *et al.*, 2015). Relative performance was obtained by dividing the germination response of each population by the mean value of the 14 populations thus obtaining a population rank. This approach allows investigating if germination sensitivity is explained by different local environmental conditions.

## RESULTS

Germination response of fresh and after-ripened seeds across multiple temperature treatments was affected by temperature, temperature regime and after-ripening period with a particularly strong positive effect of alternating regime and after-ripening (see Supplementary Data Table S2). Also, we found no significant association between germination response and seed mass (χ^2^ = 1.70, *P* = 0.19) thus suggesting that maternal provisioning is unlikely to strongly influence germination response.

### Among-population variation in germination sensitivity at extreme temperatures

**Fig. 2.**
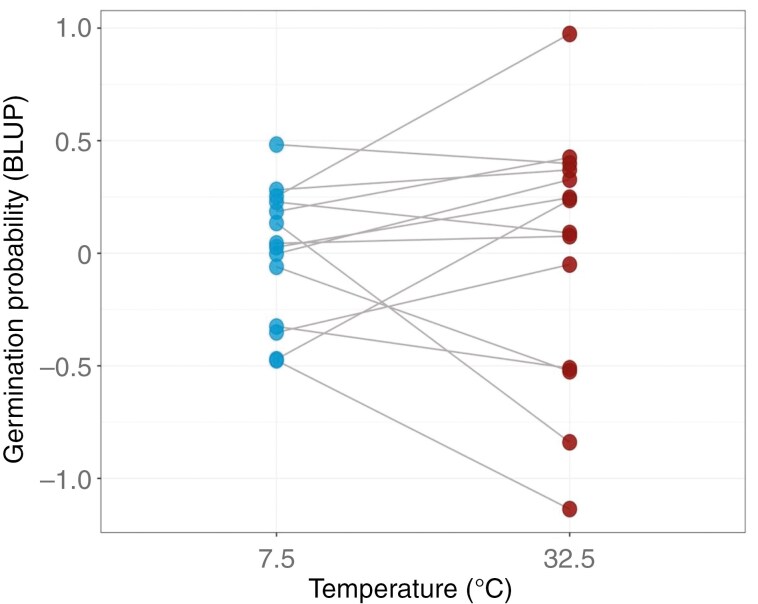
Predicted germination response (Best Linear Unbiased Predictor; BLUP) to extreme temperatures for the 14 populations of *Brassica incana*. Dots represent the germination probability for each population at each temperature extreme. Different slopes among populations suggest that populations perform differently at the two temperature extremes.

Germination response of after-ripened seeds to extreme temperatures was variable among the populations but with most populations exhibiting a good germination response to both cold and warm conditions (Supplementary Data Fig. S3). Also, we observed a positive correlation trend between the cold and the hot extreme thus suggesting that populations performing well at one extreme also perform well at the other extreme (Table 1). However, the correlation coefficient lower than 1 suggests some level of independence between the two temperature conditions and thus that populations often perform differently at hot and cold extremes (Table 1; Fig. 2). Among-population variance in germination response was comparable between the two extremes with variance at the hot extreme being only slightly higher, thus highlighting overall a similar expression of variance across the treatments (Table 1). Analysis carried out using the *glmmTMB* package showed a non-significant interaction between population and temperature, a weak positive correlation between temperature extremes and a similar expression of variance at the two extremes, as already obtained through the Bayesian approach (Tables S3 and S4).

**Table 1.** Covariance matrix for mean germination response of 14 *Brassica incana* populations. The diagonal contains the variance in germination response at each extreme temperature (values in bold). Covariances between temperatures are presented below the diagonal, and correlations between temperatures are presented above the diagonal. Numbers in parentheses denote 95 % HPD intervals (Bayesian credible highest probability density). Correlations are significant in a Bayesian framework when greater than 90 % of the posterior distribution does not overlap zero.

	7.5 °C	32.5 °C
7.5 °C	**0.8 (0.10, 1.99)**	0.2 (−0.67, 1)
32.5 °C	0.18 (−0.42, 1.14)	**1.09 (0.05, 2.32)**

### Within-population variation in germination sensitivity at extreme temperatures

**Fig. 3.**
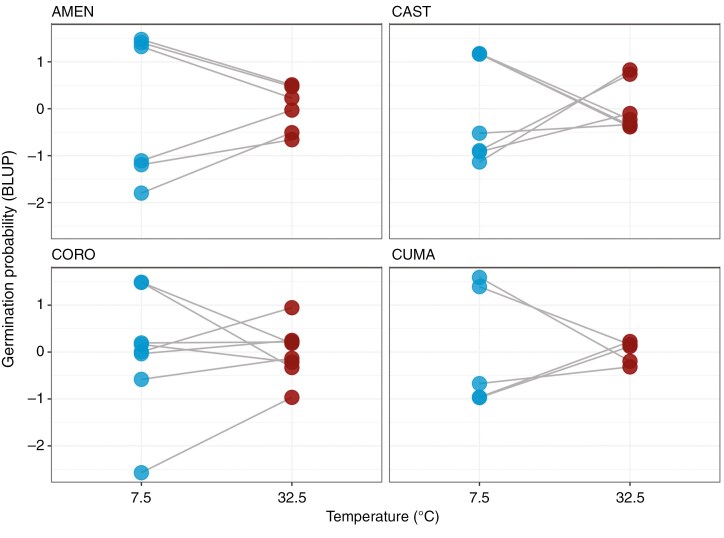
Predicted germination response (Best Linear Unbiased Predictor; BLUP) to extreme temperatures for each maternal seed family of *Brassica incana* populations with at least five maternal families (*n*_populations_ = 4). Dots represent the germination probability for each maternal family at each temperature extreme. Different slopes among maternal families suggest that maternal families perform differently at the two temperature extremes.

**Fig. 4.**
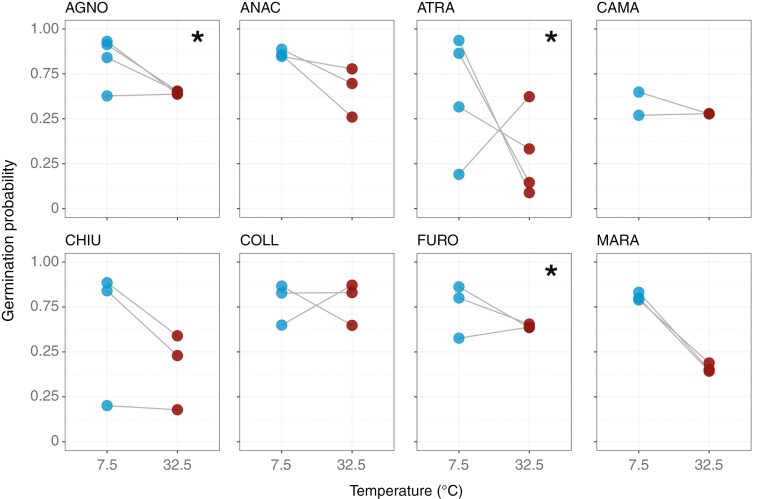
Predicted germination response to extreme temperatures for each maternal seed family of *Brassica incana* populations with fewer than five maternal families (*n*_populations_ = 8). Dots represent the germination probability for each maternal family at each temperature extreme. Different slopes among maternal families suggest that maternal families perform differently at the two temperature extremes. Asterisks (*) represent the tests where populations show trade-offs or changes in variance between cold and hot extremes.

Analyses performed among maternal families showed different mechanisms acting within the populations. Specifically, according to the Bayesian analyses, two populations (CAST and CUMA) showed a negative, although not significant, correlation between the two temperature extremes, suggesting that maternal families respond differently by showing a trade-off in germination performance between the cold and the hot extreme (Table 2; Fig. 3). The other two populations with more than five maternal families (AMEN and CORO), instead, showed a positive correlation between the treatments hence suggesting that maternal families have the same germination response at the two extremes (Table 2; Fig. 3). However, as previously described for the among-population comparison, all the correlation coefficients were lower than 1 and, in one population (CORO), we observed values near zero suggesting that different maternal families behave highly differently at the two extreme temperatures. All the populations showed an among-family variance much greater in the cold than in the hot extreme with a particularly high difference between the treatments in two populations (AMEN and CUMA). This change in variance between the treatments is also a proxy for G × E and suggests a higher adaptive potential at lower temperatures. Analysis carried out using the *glmmTMB* package showed for all the populations significant G × E interactions and the results were consistent with those of the Bayesian models (Supplementary Data Tables S3 and S4). For populations with fewer than five maternal families, we found a significant G × E, observed as a trade-off in germination response between hot and cold extremes, in one population (ATRA; Fig. 4). In two populations (AGNO and FURO), we found a significant G × E, observed as a greater variance at the cold extreme compared to the hot extreme (Fig. 4). In the five remaining populations (ANAC, CAMA, CHIU, COLL and MARA), instead, we did not find significant G × E patterns. However, we observed a different level of among-family variance at the two extremes with most populations showing a higher variance at the cold extreme compared to the hot extreme and only one population (ANAC) showing the opposite trend (Fig. 4).

**Table 2.** Covariance matrix for germination response within each *Brassica incana* population (maternal families *n* > 5). The diagonal contains the variance in germination response at each extreme temperature (values in bold). Covariances between temperatures are presented below the diagonal, and correlations between temperatures are presented above the diagonal. Numbers in parentheses denote 95 % HPD intervals (Bayesian credible highest probability density). Correlations are significant in a Bayesian framework when greater than 90 % of the posterior distribution does not overlap zero.

Population ID		7.5 °C	32.5 °C
AMEN	7.5 °C	**30.85 (0.60, 92.46)**	0.13 (−0.74, 1)
	32.5 °C	1.3 (−7.81, 15.69)	**7.88(0, 14.94)**
CAST	7.5 °C	**19.77 (3.45, 49.37)**	−0.13 (−0.99, 0.46)
	32.5 °C	−1.09 (−10.02, 6.36)	**8.55 (1.02, 18.25)**
CORO	7.5 °C	**18.69 (2.10, 49.45)**	0.07 (−0.78, 0.99)
	32.5 °C	0.93 (−6.55, 8.09)	**6.51 (0, 15.81)**
CUMA	7.5 °C	**24.16 (0.56, 73.02)**	−0.02 (−0.79, 0.68)
	32.5 °C	−0.19 (−22.08, 14.86)	**5.15 (0.14, 31.39)**

### Association between local climate and germination response to extreme temperatures

**Fig. 5.**
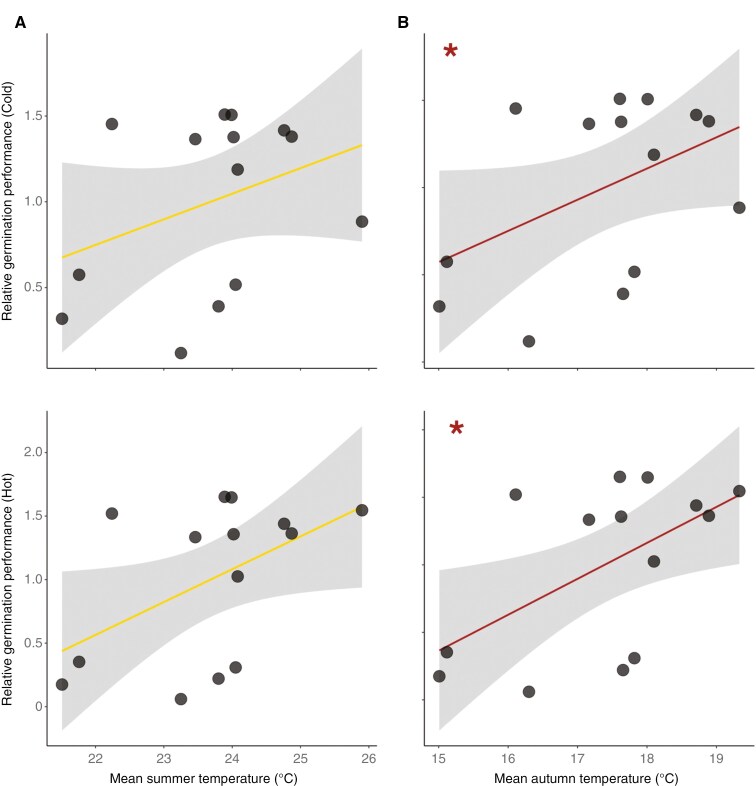
Association between local mean summer temperature (A) and mean autumn temperature (B), and relative germination response at cold and hot extremes for the 14 *Brassica incana* populations tested using linear regression. *0.01** **< *P* ≤ 0.05.

By investigating the relationship between local climate and germination sensitivity at extreme temperatures, we found a positive association between mean autumn temperature and relative germination performance under both the extreme temperatures thus suggesting that populations experiencing higher mean autumn temperatures have a higher ability to germinate both under cold and hot extreme conditions (*β* = 0.262, *P* = 0.053; *β* = 0.266, *P* = 0.035; Fig. 5A). Mean summer temperature showed a similar positive trend when associated with relative germination performance at cold and hot extremes (*β* = 0.149, *P* = 0.218; *β* = 0.259, *P* = 0.069; Fig. 5B).

## DISCUSSION

Mechanisms underlying plant species ability to face extreme conditions are often the result of a complex interplay between mechanisms acting among and within the populations, yet few studies have simultaneously investigated these factors in natural populations. Here, we quantified the response under extreme thermal conditions of the Mediterranean cliff species *B. incana* by estimating the among- and within-population variance in the seed germination response of multiple populations exposed to thermal conditions outside their local environment.

We observed a remarkable variation in germination response of after-ripened seeds with some populations showing a high performance at both cold and hot extremes and others showing a limited ability to germinate under such conditions (Table 1; Fig. 2). This variation might, however, also reflect an among-population difference in seed longevity (Hay *et al.*, 2022). In contrast to previous studies (e.g. Nussey *et al.*, 2005; Bell and Gonzalez, 2009; Chevin *et al.*, 2013; Walter *et al.*, 2020), we did not detect a significant G × E interaction in response to extreme conditions, which would have suggested a trade-off in germination response between the two extremes. However, the weak positive correlation obtained in our Bayesian analyses suggests some level of independence between the temperature conditions and thus that populations behave differently at the two extremes. Interestingly, we also observed a slightly higher among-population variance at the hot extreme compared to the cold extreme which could be indicative of local adaptation to higher temperature. Furthermore, we cannot discard the hypothesis that a significant G × E can be found by exposing seeds to more extreme temperatures or by sampling seeds across years accounting for potential within-population variation due to year-to-year variation in seed maturation conditions. Nevertheless, our study was designed to estimate other sources of variation at the species level (accounting for multiple populations) and at the population level (accounting for different maternal families).

In our study system, we observed that populations differ mainly according to their overall ability to germinate under extreme temperatures. A similar result was observed in an experiment where different populations of an annual plant were grown under moist and drought conditions and showed a similar level of fitness regardless of the treatment (Heschel *et al.*, 2004). Interestingly, Matesanz and Ramírez‐Valiente (2019), through a comprehensive literature review, revealed that although plant population differentiation is highly common, it is often neglected when predicting species response under climate change. This evidence, also supported by the high among-population divergence observed in this study, confirms the importance of examining multiple populations to identify those exhibiting a better performance under novel conditions, thereby aiding in maintaining the resilience of the species (Cochrane *et al.*, 2015; Barga *et al.*, 2017; Matesanz and Ramírez‐Valiente, 2019). This is particularly true for Mediterranean plants with highly fragmented distributions, such as the investigated *B. incana*, since their chance to migrate in response to climate change is limited and thus *in situ* responses play a crucial role (Benito Garzón *et al.*, 2019; Muffler *et al.*, 2021).

Mean population response can hide plastic and genetic mechanisms acting within the populations and understanding these mechanisms is critical for identifying the ability of populations to persist under novel environments (Donohue and Schmitt, 1998; Uller *et al.*, 2013; Cochrane *et al.*, 2015). Here, we used an experimental design where different maternal families were tested, to disentangle the mechanisms shaping the average level of germination ability of each population under extreme temperatures. Our design allows for reducing the amount of genetic variance compared to a design involving seeds with unknown parental origin and is also widely used in natural populations where controlled crosses are difficult to carry out (Falconer and Mackay, 1996; Gauzere *et al.*, 2013 and references therein).

The analyses performed within the 12 *B. incana* populations showed a high variation in germination response under extreme temperatures. Accordingly, several studies reported high within-population variability in germination traits (e.g. Cohen, 1966; Liyanage and Ooi, 2015; Friedman *et al.*, 2019) with some studies supporting the hypothesis that maternal effects had a key role in driving this variation (e.g. Carta *et al.*, 2016; Muffler *et al.*, 2021) and others showing that variation was mainly attributable to additive genetic effects (e.g. Zas and Sampedro, 2015). Our experimental design was based on field-collected maternal families, and we thus accounted for the role of maternal provisioning in shaping such variations by testing the association between seed mass and germination response. Although we cannot discard the role of maternal effects, the lack of a significant association suggests that maternal provisioning is unlikely to strongly influence within-population variation in *B. incana*.

Interestingly, the level of among-family variance was variable among populations and mechanisms underlying these variations were different (Table 2; Figs 3 and 4). Specifically, two populations (CAST and CUMA) showed a weak, although not significant, negative correlation between the two treatments thus suggesting a trade-off in germination response between the hot and the cold extreme (Table 2). The other two populations with more than five maternal families (AMEN and CORO), instead, showed a weak positive correlation between the treatments. However, in all the Bayesian models, we obtained correlation coefficients near zero thus suggesting that different maternal families show highly different responses to the two extreme temperatures (Table 2; Fig. 3). Also, in all the populations, we observed G × E as a greater among-family variance at the cold extreme compared to the hot extreme (Table 2; Fig. 3). Similar results were obtained for populations with fewer than five maternal families. Specifically, one population (ATRA) exhibited a significant G × E as a trade-off in germination response between the two extreme temperatures (Fig. 4), while in two populations (AGNO and FURO), G × E was verified by a change in variance between the two extreme temperatures with both populations showing a greater among-family variance at the cold extreme compared to the hot extreme (Fig. 4). In all the other populations, although we did not detect significant G × E interactions, we observed a highly variable response to the two temperature extremes. This lack of significance might, however, be explained by the low number of maternal families included in the analysis.

Changes in variance between extreme temperatures, detected in some populations, suggest that germination is more predictable under specific temperature conditions. If populations exhibit high among-family variance under novel, extreme temperatures, they will be more likely to persist under those conditions, and their ecological resilience will increase. Interestingly, the higher among-family variance detected at lower temperatures in most populations contrasts sharply with the higher among-population variance at higher temperatures. This result suggests that some populations might be locally adapted to higher temperatures, but their adaptive potential is probably higher at the cold extreme. Differently, populations exhibiting G × E as a trade-off in germination response with maternal families performing significantly better at different extreme temperatures are likely to be favoured compared with populations producing seeds that germinate predominantly in one condition or the other, especially under unpredictable environmental conditions typical of the Mediterranean climate (Giménez-Benavides *et al.*, 2005; Feng *et al.*, 2022; Mattana *et al.*, 2022). Previous studies demonstrated how environmental heterogeneity could maintain genetic variation within populations by imposing variable selection on critical plant life traits, such as flowering time (Mojica *et al.*, 2012; Lee *et al.*, 2016; Troth *et al.*, 2018). By analysing a population of *Mimulus guttatus* exposed to changes in water availability across the flowering seasons, Troth *et al.* (2018) found that in years with abundant moisture, late-flowering individuals are favoured through fecundity selection, while in drought years earlier flowering plants are favoured. These contrasting selective pressures enable populations to maintain allelic variation for those traits being under selection. Alternatively, population responses may not depend upon the environment in which they are tested thus revealing a weak or even absent G × E (Barga *et al.*, 2017), a pattern also found in our study system. However, even in populations with no significant G × E patterns, we found different responses among maternal families between the two extreme temperatures. Overall, these findings suggest that *B. incana* populations harbour considerable developmental flexibility in germination response under novel environmental conditions.

To investigate if local climate explained among-population differences in environmental sensitivity, we compared the relative population performance at extreme temperatures to the temperature experienced by each population during their germination period. Interestingly, we observed that populations experiencing higher temperatures (i.e. strictly Mediterranean populations) also showed a higher sensitivity (i.e. higher performances at both extreme temperatures) compared to those from colder environments (Fig. 5). Our results are in line with previous studies reporting that germination response was explained by different local environmental conditions (e.g. Carta *et al.*, 2016; Braga *et al*., 2017; Walter *et al*., 2020). Particularly, as in our study, Carta *et al.* (2016) found that populations from the warmest sites showed the highest germination under all the investigated treatments. Our findings suggest that the highly seasonal dry and hot climate, typical of the Mediterranean region, might select for a higher plasticity in germination response and thus a higher sensitivity. Such plasticity might be interpreted as a particularly efficient survival strategy for species growing in the Mediterranean environment (Giménez-Benavides *et al.*, 2005; Carta *et al.*, 2022). Indeed, the ability to face extreme environmental conditions can play a key role in shaping the performance of populations under climate change, particularly in the Mediterranean where a higher sensitivity can confer them an advantage to cope with the predicted increase in aridity and climatic heterogeneity.

## CONCLUSIONS

By investigating the germination response to extreme conditions across 14 populations of the Mediterranean species *B. incana*, we showed that environmental sensitivity at early plant life cycle phases can differ highly among and within populations. Also, we demonstrated how among-population differences at extreme conditions might be explained by local climate. Our results suggest that strictly Mediterranean populations have a greater potential to face future temperature extremes due to their ability to germinate under a wide range of environmental conditions and can thus be crucial to promote species ecological resilience under a climate change scenario.

## SUPPLEMENTARY DATA

Supplementary data are available at *Annals of Botany* online and consist of the following.

Table S1: Sampling locations of the 14 populations of *Brassica incana.* Methods S1: Estimation of the overall effects of temperature, temperature regime and after-ripening on germination response of *Brassica incana* populations using binomial generalized mixed models with Bayesian estimation. Table S2: *MCMCglmm* output for the Bayesian model testing the effect of temperature (T), regime (R) and after-ripening (AR) on germination response in *Brassica incana* populations. Table S3: Mixed linear models testing for significant genotype-by-environment interactions in germination response among different *Brassica incana* populations and different maternal families within the populations. Table S4: Covariance matrix for mean germination response of *Brassica incana* populations and within each *B. incana* population (maternal families *n* > 5). Figure S1: Germination proportion of *Brassica incana* under constant and alternating temperature regimes in fresh (pink bars) and after-ripened (blue bars) seeds. Error bars represent 95 % binomial confidence intervals. Figure S2: Mean autumn temperature (A) and mean summer temperature (B) extracted from the 14 *Brassica incana* populations at 1.25 arc-min resolution. Figure S3: Mean germination proportion across all populations of *Brassica incana* under extreme temperatures in after-ripened seeds. Error bars represent 95 % binomial confidence intervals.

mcae172_suppl_Supplementary_Tables_S1-S4_Methods_S1

mcae172_suppl_Supplementary_Figures_S1

mcae172_suppl_Supplementary_Figures_S2

mcae172_suppl_Supplementary_Figures_S3
