# Supplementary material for: Among- and within-population variation in germination response shapes ecological resilience in the Mediterranean cliff species Brassica incana
Source: Ann Bot. 2024 Oct 14;135(3):451–62. doi: 10.1093/aob/mcae172 (PMC11897589; doi:10.1093/aob/mcae172)
Supplement: mcae172_suppl_Supplementary_Tables_S1-S4_Methods_S1 [file mcae172_suppl_supplementary_tables_s1-s4_methods_s1.docx]

**Methods S1**

To estimate the overall effects of temperature, temperature regime, and after-ripening, as well as their interactions, on germination response, we fitted binomial generalized mixed models with Bayesian estimation implemented in the R package MCMCglmm using data collected both on fresh and after-ripened seeds by implementing the following equation:

*y_ijklmno_ = T_i_* ✕ *R_j_ + T_i_* ✕ *A_k_ + p_l_ + m_m_ + d_n_ + e_o(ijklmn)_*

where *T_i_* is the average incubation temperature, treated as a categorical variable, *R_j_* is the temperature regime, *A_k_* is the after-ripening, all included as fixed effects, and *p_l_*, *m_m_* and *d_n_* are the population, the maternal family and the petri dish, respectively, all included as a random factor, and *e_o(ijklm)_* is the model error. Variables were scaled to get comparative effect sizes.

We implemented the equation using 3,000,000 Markov Chain Monte Carlo (MCMC) sampling iterations, with a burn-in period of 300,000 iterations and a thinning interval of 1,000 iterations. We checked model convergence by confirming that effective sample sizes exceeded 85% of the specified number and that autocorrelation values did not exceed 0.05. We used an uninformative parameter-expanded prior, which we checked by changing the scale parameter and ensuring there was no effect on the estimation of the posterior distribution.

**Table S1.** Sampling locations of the 14 populations of *Brassica incana.*

| **Locality** | **Population ID** | | **Latitude** | **Longitude** |
| --- | --- | --- | --- | --- |
| Agnone | AGNO | 37.3163 | | 15.0997 |
| Ischia - Lacco Ameno | AMEN | 40.7516 | | 13.8966 |
| Anacapri | ANAC | 40.5457 | | 14.2232 |
| Atrani | ATRA | 40.6367 | | 14.6100 |
| Camaldoli | CAMA | 40.8551 | | 14.2077 |
| Castellammare | CAST | 40.6822 | | 14.4398 |
| Valico di Chiunzi | CHIU | 40.7190 | | 14.6191 |
| Colli | COLL | 40.6196 | | 14.4474 |
| Coroglio | CORO | 40.7983 | | 14.1758 |
| Cuma | CUMA | 40.8504 | | 14.0499 |
| Ischia - Epomeo | EPOM | 40.7300 | | 13.8953 |
| Furore | FURO | 40.6144 | | 14.5482 |
| Maratea | MARA | 40.0421 | | 15.6525 |
| Vietri di Potenza | POTE | 40.5709 | | 15.5202 |

**Table S2.** *MCMCglmm* output for the Bayesian model testing the effect of Temperature (T), Regime (R), and After-ripening (AR) on germination response in *Brassica incana* populations. Post.Mean is the mean parameter estimate calculated from the posterior distributions; lower and upper credible intervals (CI) are provided along with the probability of the value of the predictor differing from zero (in bold when significant).

|  | **Post.Mean** | **Lower 95% CI** | **Upper 95% CI** | **eff.samp** | **pMCMC** | Effect |
| --- | --- | --- | --- | --- | --- | --- |
| T(20 °C) | -0.210 | -0.728 | 0.216 | 2700 | 0.387 | Fixed |
| T(25 °C) | -0.563 | -0.943 | -0.135 | 2700 | **0.016** | Fixed |
| R | 0.991 | 0.198 | 0.999 | 2700 | **< 0.001** | Fixed |
| AR | 0.991 | 0.245 | 0.995 | 2700 | **< 0.001** | Fixed |
| T(20°C): R | -0.342 | -0.789 | 0.145 | 2700 | 0.159 | Fixed |
| T(25°C): R | -0.681 | -0.916 | -0.201 | 2700 | **0.004** | Fixed |
| T(20°C): AR | 0.450 | -0.014 | 0.915 | 2700 | 0.056 | Fixed |
| T(25°C): AR | 0.427 | -0.034 | 0.874 | 2700 | 0.072 | Fixed |
| Population | 0.918 | 0.267 | 1.910 | 2700 |  | Random |
| Family | 0.374 | 0.001 | 1.188 | 2700 |  | Random |
| Obs | 1.659 | 0.001 | 3.183 | 2416 |  | Random |
| units | 1.454 | 0.118 | 3.172 | 2437 |  | Residual |

**Table S3.** Mixed linear models testing for significant genotype-by-environment interactions in germination response among different *Brassica incana* populations and different maternal families within the populations. To determine significance of the improved fit of each model, likelihood ratio tests were performed (p value of test displayed; in bold when significant).

|  | **Model** | **Likelihood ratio test (p value)** |
| --- | --- | --- |
| B. incana | y ~ T + (1\|Population) | - |
|  | y ~ T + (T - 1\|Population) | 0.435 |
| AMEN | y ~ T + (1\|Family) | - |
|  | y ~ T + (T - 1\| Family) | **0.021** |
| CAST | y ~ T + (1\| Family) | - |
|  | y ~ T + (T - 1\| Family) | **0.001** |
| CORO | y ~ T + (1\| Family) | - |
|  | y ~ T + (T - 1\| Family) | **0.012** |
| CUMA | y ~ T + (1\| Family) | - |
|  | y ~ T + (T - 1\|Population) | **0.005** |

**Table S4.** Covariance matrix for mean germination response of *Brassica incana* populations and within each *Brassica incana* population (maternal families *n*>5). The diagonal contains the variance in germination response at each extreme temperature (values in bold). Covariances between temperatures are presented below the diagonal, and correlations between temperatures are presented above the diagonal. Numbers in parentheses denote 95% confidence intervals.

|  |  | 7.5 °C | 32.5 °C |
| --- | --- | --- | --- |
| *B. incana* | 7.5 °C | **0.57 (0.21, 1.56)** | 0.36 (-0.80, 0.93) |
|  | 32.5 °C | 0.23 (-0.35, 1.97) | **0.74 (0.40, 1.36)** |
| AMEN | 7.5 °C | **28.27 (1.5, 50.67)** | 0.14 (-0.45, 0.67) |
|  | 32.5 °C | 1.11 (-6.9, 14.32) | **7.97 (0.56, 15.88)** |
| CAST | 7.5 °C | **19.13 (3.9, 32.87)** | -0.09 (-1, 0.88) |
|  | 32.5 °C | -0.05 (-7.03, 5.12) | **8.02 (1.92, 15.33)** |
| CORO | 7.5 °C | **17.63 (1.62, 38.51)** | 0.07 (-0.88, 0.78) |
|  | 32.5 °C | 0.43 (-5.44, 8.19) | **5.11 (0.11, 17.29)** |
| CUMA | 7.5 °C | **23.98 (1.28, 54.01)** | -0.02 (-0.76, 0.66) |
|  | 32.5 °C | -0.18 (-17.94, 13.05) | **5.19 (0.36, 12.49)** |
