# Supplementary figures and images for: Among- and within-population variation in germination response shapes ecological resilience in the Mediterranean cliff species Brassica incana
Source: Ann Bot. 2024 Oct 14;135(3):451–62. doi: 10.1093/aob/mcae172 (PMC11897589; doi:10.1093/aob/mcae172)

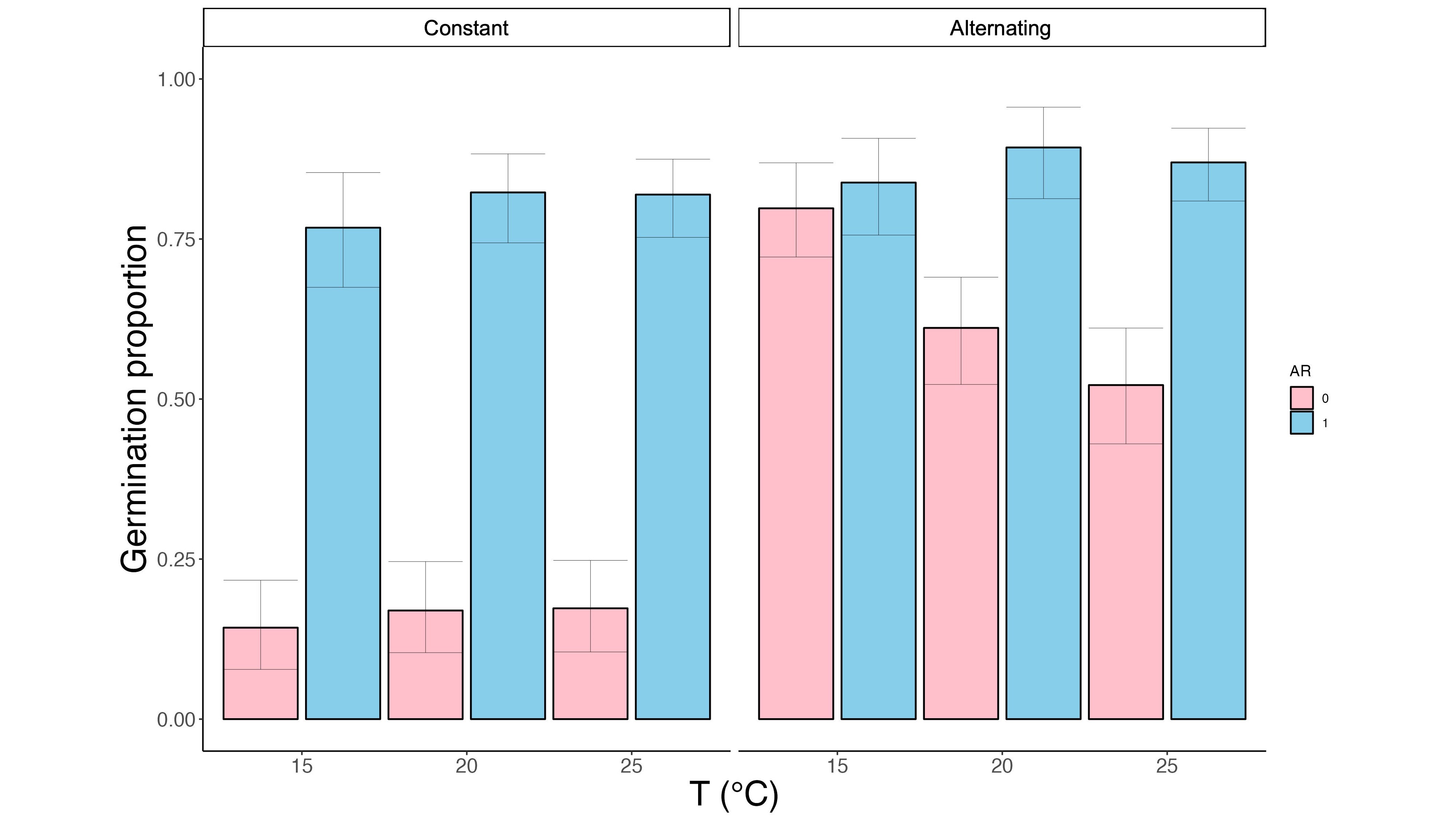

Supplement: mcae172_suppl_Supplementary_Figures_S1 [file mcae172_suppl_supplementary_figures_s1.jpeg]

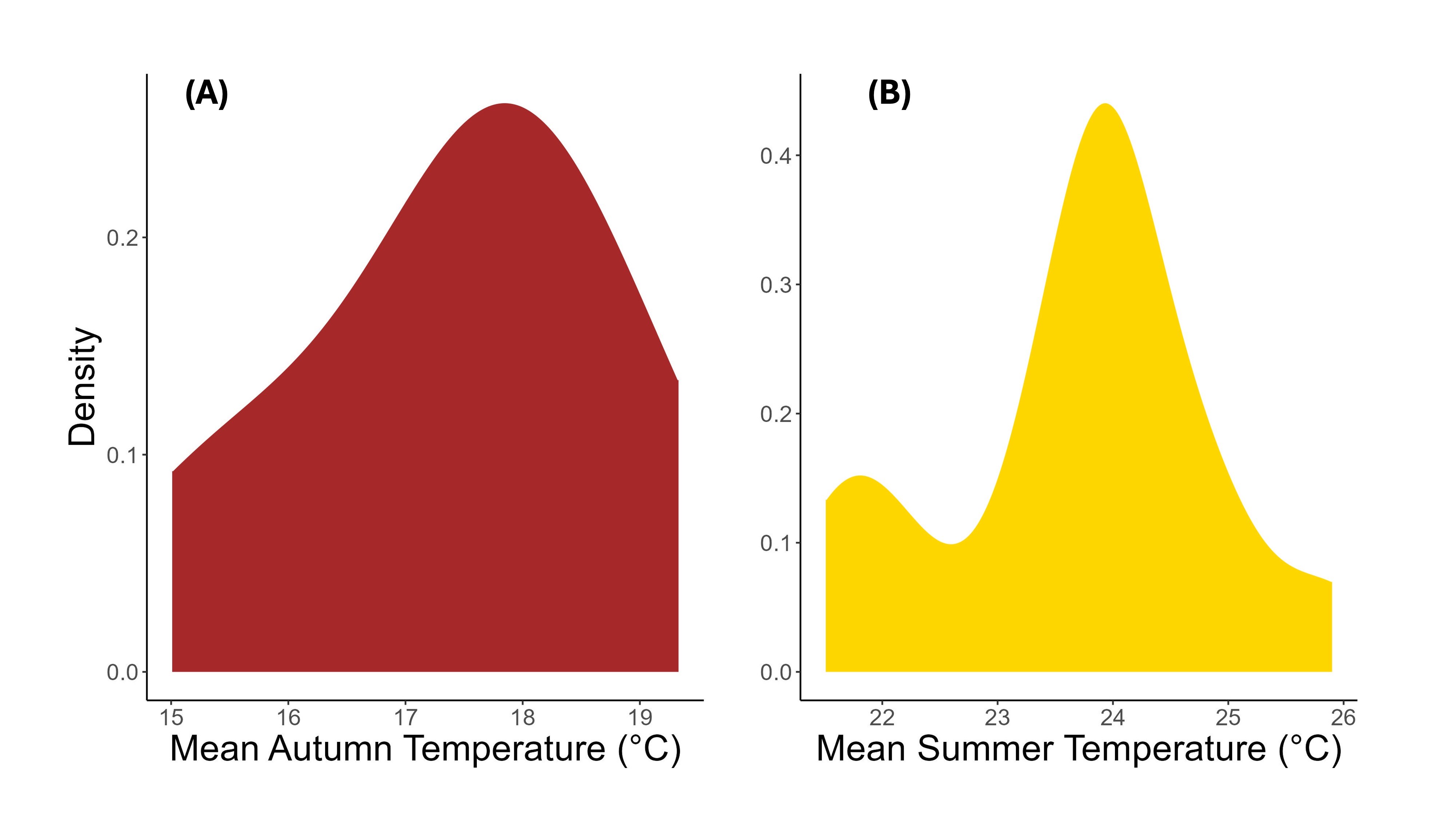

Supplement: mcae172_suppl_Supplementary_Figures_S2 [file mcae172_suppl_supplementary_figures_s2.jpeg]

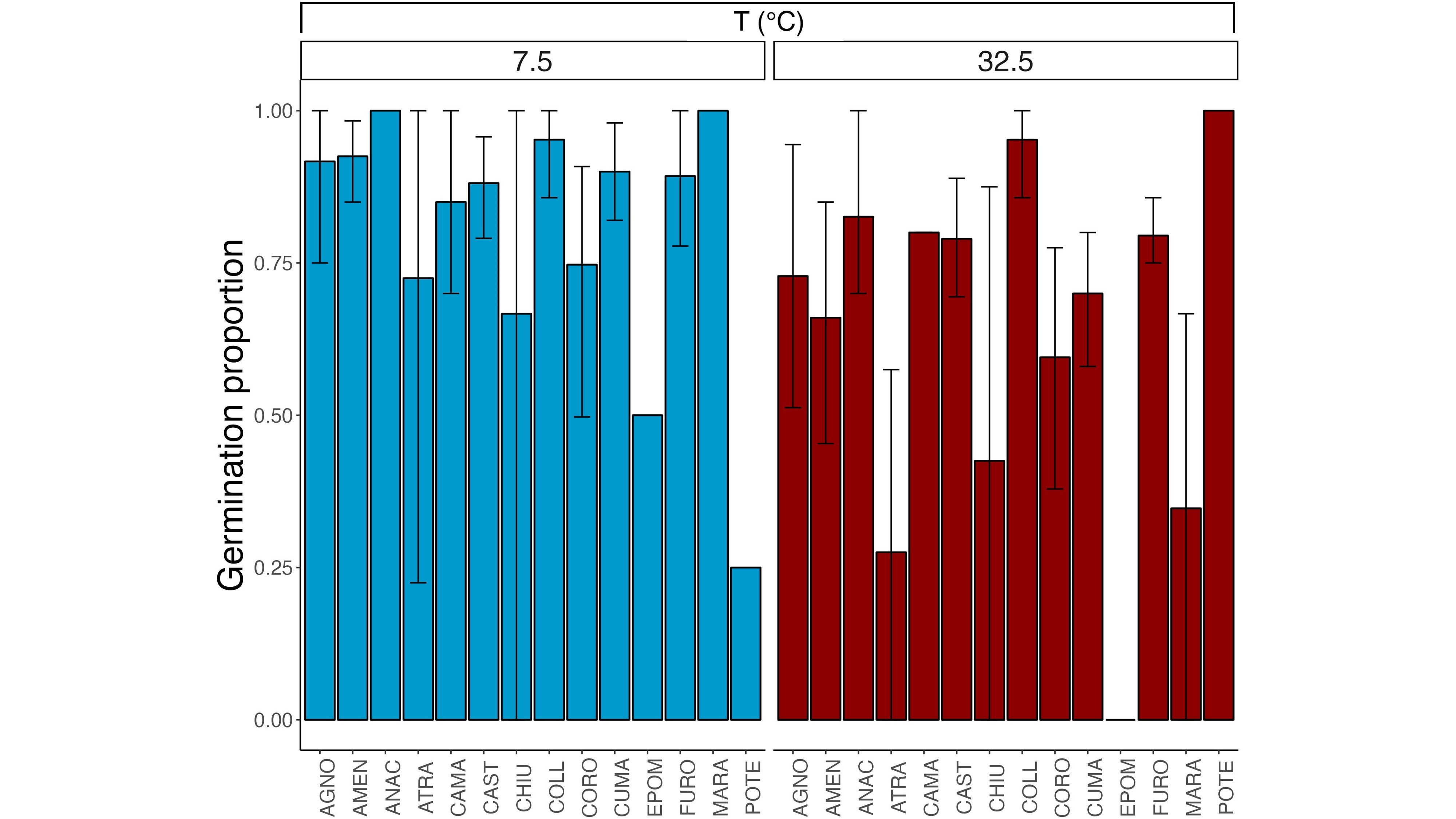

Supplement: mcae172_suppl_Supplementary_Figures_S3 [file mcae172_suppl_supplementary_figures_s3.jpeg]
